# Lectures on Scarlet Fever

**Published:** 1851-04

**Authors:** Caspar Morris

**Affiliations:** Late Lecturer on Practical Medicine in the Philadelphia Medical Institute


### MEDICAL EXAMINER,

AND

### RECORD OF MEDICAL SCIENCE.

NEW SERIES.—NO. LXXVI.—APRIL, 1851.

### ORIGINAL COMMUNICATIONS.

*Lectures on Scarlet Fever*. By Caspar Morris, M. D., late Lecturer on Practical Medicine in the Philadelphia Medical Institute.

### Lecture III.

As yet every step taken in the process of investigation into the nature and habits of the cause of this disease, leads further from certainty as to its character and the laws by which it is governed. Though prevailing most frequently during the changeable weather either in the spring or autumn, and extending itself through the winter, in this respect manifesting its affinity to other febrile eruptive diseases with which it is sometimes associated, measles, erysipelas, and varicella, it is much more common to meet with isolated cases of it than of them which can not be traced to any infection, and which fail to propagate the disease in others who are brought most closely in connexion with it.

I have seen quite recently, during the intense heat of July, one well marked and fatal case, in a large family of children, without spreading to any other members, though no attempts at isolation were made. Similar cases are familiar to every physician. The access to the chamber was as free, and the visits of the other children were as frequent, as the warmest affection could dictate. The subject was the youngest child, and an only daughter, and therefore the least exposed to external influences; it were vain to attempt any solution of the question from whence the miasm was derived which gave rise to this case. Such are of frequent occurrence, and if received without any investigation of the circumstances which modify them, and without any regard to others of an opposite tendency, would shake the faith of the most devoted believer in the contagious character of the disease. There is, however, some peculiar want of susceptibility to the impression of morbid poisons in this family, as they had escaped with equal impunity, a few months previously, when varioloid was brought into the house by a relative, taken into it, without the adoption of any precautionary measures, from a boarding house where he had sickened with that disease.

Some eminent teachers of medical science are disposed to solve the difficulty growing out of the frequent observation of such cases, in a very summary manner. Whilst they admit them to be characterized by all the symptoms of scarlet fever, so that we cannot in any way distinguish them from that disease, they think the mere absence of the power of propagation, and the inability to trace them to any connexion with a previous case or epidemic influence, sufficient reason to consider them an entirely distinct disease. Such is the opinion of Professor Mitchell, and such appears to be that of Dr. Chapman, who, in his lectures on exanthematous fevers, says, "Moreover I have repeatedly met with in the winter, and uniformly, I think, when snow is on the ground, an affection bearing a still closer resemblance to scarlatina. Children acquire it, mostly, or perhaps exclusively, by becoming heated from playing in the snow, and then suddenly chilled. The collapse is often very considerable, followed by febrile reaction, much cerebral disturbance, delirium, stupor, and sometimes convulsions, sore throat, nausea and vomitings, and by an eruption so scarlatinous in its aspect, that it may readily be mistaken for that disease. Nor is it scarcely less fatal. Death, I have known to take place from it in a few hours, from an inability to arouse the system out of its torpor, or by convulsions." You are well aware of the high estimate I place on the quickness of perception, clear judgment, and perfect accuracy of of Professor Chapman, and long observation has given me entire confidence in the correctness of his diagnosis; yet even the weight of his authority cannot lead me to doubt the dependence of the cases he thus describes upon the miasm which produces scarlet fever. While we know, on the one hand, that the causes of other diseases may lie long dormant in the system, only waiting for exciting circumstances to develope their activity, and, on the other, that even the most contagious diseases occur occasionally as sporadic cases, it appears to be more in accordance with analogy to consider such cases as scarlet fever, failing to diffuse itself, from some unknown cause, than to erect a new form of disease distinguished from scarlet fever by no symptom, but this one negative characteristic.

In the month of December, 1832, the disease appeared among the inmates of the House of Refuge for juvenile offenders. It was prevailing in the city at that period, as will be proven by reference to the table furnished by Dr. Emerson which exhibits the mortality of that year as the highest in the ten years from 1830 to 1840. The discipline of this institution is such as secludes the children almost entirely from communication with persons beyond its walls. This might be supposed to afford an unusually valuable field for observation on its introduction and mode of diffusion. The difficulty we encounter, however, in determining the manner in which it was introduced into the institution, is one which exhibits the obscurity in which the question is enveloped. I was at the time the attending Physician, and made a daily record of every case of sickness requiring my care, in a book kept in the institution for that purpose. A few cases of sore throat had been noted; one of erysipelas, and several of varicella, all on the boys' side of the institution, within the fortnight which preceded the breaking out of scarlet fever.

The first well marked case, however, occurred in the person of a visitor to the family of the matron, thus throwing a strong shade of uncertainty on the question of origin. The second was that of a sister of the first; and the first cases among the permament inmates occurred at the time of the convalescence of the first patients, in the matron's family. Had no cases of sore throat among the children preceded the first well developed case, the presumption that the disease had been introduced by her from without, would have been strong, especially as the apartments occupied by the matron's family, were in that part of the premises appropriated to the females, and it was among the females the disease first diffused itself. The buildings are extensive, surround a very large enclosure, and are so arranged as to effect a separation of the sexes at *all times*. The disease spread rapidly among the girls, five cases occurring simultaneously on the 25th of December; and one boy on the 29th of the same month; two other boys were taken on the 11th of January 1833. On the 19th a case of varicella was presented among the boys, followed by several others within the following week. Scarlet fever and sore throat without eruption, continued to prevail among both boys and girls till the end of March, amounting in all to sixty cases, without one fatal termination. If no case of sore throat with fever among the children had preceded the sickness of the visitor, this history would have been very decidedly favorable to the idea of the dependence of the disease on contagion; and this would have been strengthened by the manner in which it spread to her immediate attendants, and the inmates of the apartment in direct communication with her sick room. The interval also between the first case and those which followed, was such as is most conformable to the idea of its contagious character; and this is confirmed by the number then seized simultaneously. But any positive decision as to the mode of introduction, is forbidden by the previous prevalence of sore throat, with fever, among the children. MM. Rilliet and Barthez, in recording their observation of the disease, in the wards of the Hospitals for sick children in Paris, appear, on the contrary, to be able to trace every period of its prevalence in that institution, that came under their notice, to the introduction of a patient suffering with it, from whom it spread to the children who had been brought in with other diseases.

Among the circumstances which favor the idea of some influence which is propagated in the person of the sick, and thus diffused from one individual to another, no slight degree of importance is due to the fact that it is said to be unknown in those distant colonies settled by Europeans, the voyage to which is so long that the influence of the specific poison is exhausted before the arrival of the vessel in which it might be transported to their shores. Thus it is said to be unknown in the thickly peopled and civilized colonies of Australia and Van Dieman's Land ; and Dr. Gregory asserts that there is no record of its prevalence in the Indian possessions of Great Britain. If direct contagion, or the transportation of some "materies morbi," eliminated in the progress of a case, be necessary to act as a ferment in diffusing the disease, and its epidemic prevalence has never an independent origin, places thus far removed from the old centres of civilization may forever remain exempt; unless we adopt the extreme view of Dr. Elliotson, as quoted by Dr. Chapman, who believes the infecting cause may be retained in the apartments where the disease has once existed, a much longer period than that occupied by these voyages. He asserts that "all the children admitted into a particular ward under his care, in one of the London Hospitals, were seized with scarlet fever for two years, in consequence of a patient with the disease having been in the ward at that remote period, and this, in despite of white-washings and other cleansings." The want of any regular medical reports from those countries, leaves a doubt as to the accuracy of the statement, that scarlet fever is unknown there. The point is one well worthy of investigation, and our own new settlements on the Pacific, will afford an excellent field for careful observation.

Reasoning upon the probabilities of the case, we should be disposed to the conclusion that it would be found to exist in all parts of the world. The constituents of the atmosphere, the physical arrangements of the material creation, the nature of man himself, his tendencies and susceptibilities, have ever been the same ; it would be therefore but a natural presumption, that the diseases to which he is subject should undergo no greater modification than results from the varying habits which mark different stages of refinement or circumstances of wretchedness.

True it is, that diseases supposed to be new, have from time to time made their appearance, and diffused themselves in a manner as mysterious as scarlet fever. Black death, sweating sickness, cold plague, cholera, have each in successive ages, performed their ministry, by thinning out the teeming population of the earth, and then disappeared from observation. But the imperfect records of these visitations, which have been transmitted to us leave room for the doubt whether they have not been mere modifications of the same disease, or the revival of plagues, the records of which have escaped from the grasp of history. The great diversity in character, presented by different epidemics of of scarlet fever, when taken in connexion with the known dependence of these several forms of the disease on one common cause, appears to favor such an assumption. Were it not so entirely established by testimony beyond contradiction, who could believe that forms of disease so apparently dissimilar as those assumed by scarlet fever in its different epidemic visitations, depend on a common cause? So frequently does this disease disappear from observation in a given district, (and occasionally, as we have seen, even from large cities,) so varying are the phases in which it presents itself, and so uncertain are the traces of the mode by which it has been introduced, that until further light is thrown upon it, we must be content to believe that it does at times commence anew from the operation of unknown influences.

Far from us be the worse than pagan infidelity, which would overlook the interference of Divine Providence, in the ordering of times of sickness or of health. If heathen moralists and heathen poets could recognize the interposition of the Gods, or seek to appease the rage of an incensed Apollo, it were surely shame for those who live in the light of Revelation, to rest on second causes only. It is, however, a duty incumbent on us as members of that profession to which the community entrusts the interests of health and life, to investigate narrowly, all second causes and collateral circumstances, which may furnish occasion for the origin, or facilitate the diffusion of this, as of every other of the many diseases, which are employed as "scourges of humanity." False indeed, were that faith, alike unsound in its foundation and disastrous in its application to man's necessities, which would teach us to lay aside our reason and cease to exert our powers for self protection. If variola may not be exterminated, nor cholera driven from the pale of civilization, vaccination mitigates the horrors of the one, and cleanliness and free ventilation, abate the intensity of the other. So if scarlet fever depend for its diffusion upon direct contagion alone, we may curtail its extent by proper precautions, which are, however, onerous and inhuman, if it have no such power of propagation. I would therefore, urge upon you, to begin from the very commencement of your professional career, a series of careful records of every fact which can throw light upon this question, not only with reference to the disease now under consideration, but all others of a kindred character. It is as much the duty of the physician to foresee the coming evil, and recommend precautionary measures, as to cope with disease in its individual manifestations in the chamber of sickness. The remarks of Dr. Rush on this subject are scarcely too strong, when in speaking of yellow fever, he says, "to all natural evil, the Author of nature has kindly prepared an antidote. Pestilential fevers furnish no exception to this remark. The means of preventing them, are as much under the power of human reason and industry, as the means of preventing the evils of lightning and common fire."

To trace down from remote ages, the mere record of the early history of the disease would be an unprofitable consumption of our time, too limited already for the investigation of matters of greater importance to you. It is not uninstructive, however, to know that this same uncertainty, as to its origin and mode of extension, has marked its character in every age; and hence, before the free diffusion of knowledge, through the agency of the press, put the members of the profession in possession of more extended information as to what was passing, or had occurred beyond the sphere of their own observation, it was *frequently* reported as a *new disease.* The earliest trace of its existence which has fallen under my own notice, dates in the first century of the Christian era, and is found in one of the tragedies of Seneca, where a chorus describes the devastation of Thebes by the plague, with which that city was visited on account of the involuntary incest of Œdipus, in the following terms :—

O dira novi facies leti!
Gravior leto! Piger ignavos
Alligat artus, languor, et ægro
Rubor in vultu, maculæque caput
Sparsere leves: tum vapor ipsam
Corporis arcem flammeus urit
Multoque genas sanguine tendit.

Oculique rigent, et sacer ignis
Pascitur artus. Resonant aures,
Stillatque niger naris aducæ
Cruor: et venas rumpit hiantes.

To those of you who have retained your familiarity with classic literature, it were an ungracious act to attempt to point out the admirable adaptation of this description, to the malignant form of the disease now under consideration. No poet or philosopher of the present day could furnish a more accurate delineation in the same number of words, unless himself familiarised with the disease by personal observation. Every lineament traced by the poet, is admirably correct. The adjuration of the *new* form of mortality more dreadful than death itself; commencing with languor of the limbs, while redness appears upon the face, and light spots are diffused over the head, from whence the influence extends to the trunk, while the parts about the jaws are distended with blood, and the eyes are distorted and a red flush is diffused over the limbs, the ears disturbed by ringing sounds, a black discharge flowing from the nostrils, and gaping ulcers are formed in the passages, all are highly characteristic. But that which Seneca then considered a new disease, has been called such many times in succeeding ages, disappearing for a period of longer or shorter duration, only to reappear in one of its several types. By the earlier medical authors, whose works have been handed down to us in imperfect fragments, all epidemic diseases were concluded under the general term of Loimos or Pestilence ; the whole family of plagues was thus grouped together. Slowly, one after another was singled out for more accurate description, but it was not till the beginning of the 17th century, that scarlet fever was separately described, and then by the Spanish and Italian authors under the terms Garotilla and morbus strangulatorius, names in themselves sufficiently descriptive to designate the disease.

Even long since that period it was, by many, confounded with measles, to which fact we may ascribe the mortality of some nominal epidemics of that disease. The accounts given by these authors refer to epidemics which prevailed in the interval between 1610 and 1650, during which time it was marked by fearful mortality. How completely it had lost its formidable character when Sydenham describes its appearance in London, in 1690 to 1695, may be inferred from his assurance that it is "a disease more in name than reality." Had Fothergill's reading been confined to Sydenham's account, he might well have regarded the epidemic, which came under his notice in the same metropolis in less than a century, as a new disease. Equally great have been the subsequent changes as recorded by the European writers, to whose works I must refer you for additional information on this point, and especially to the elaborate article by Dr. Tweedie in the Cyclopedia of Practical Medicine.

The earliest record I have been able to trace of its existence on this continent, reaches back to the year 1734—5 at which time it was prevalent in America and Europe simultaneously; and so great was the mortality caused by it in Boston, that we find the number of deaths in those years raised from 458, the number of the year 1733, to 528 in '34, and 617 in '35. At the same period the interments in the burial grounds of Christ Church and St. Peters in Philadelphia, rose from 96 to 144, and a like increase occurred in the same places from a similar cause in 1742. When we recall to mind the difficulty of communication between the Colonies at that early period, and the consequent infrequency of intercourse, and reflect on the small probability that those who were sick or so recently convalescent, as to carry about them a contagious influence, could endure the fatigue and exposure necessarily incident to the only modes of conveyance then known, we shall be disposed to adopt the solution afforded by the prevalence of a wide spread epidemic influence, as the means of propagation.

There is an exceedingly interesting paper by Cadwallader Colden, Esq., of New York, published by Dr. Fothergill in the 1st volume of the "Medical Observations and enquiries by a Society of Physicians in London," dated Coldenham, in New York, October, 1753, which throws unexpected and important light on the history of the disease on this continent. The whole document is one of great merit,and deserves to be borne in mind as a favorable specimen of our earlier medical literature. Mr. Colden remarks, "I have seen it only in my own family, and in a few neighbors in the country, to whom I sometimes give advice when they cannot obtain assistance otherwise, having laid aside the practice of physic, upwards of twenty years. What I chiefly learned was from the late Dr. Douglass* of Boston, a gentleman of great skill in medicine, and an accurate observer, having corresponded with him while this distemper was frequent in the part of the country where I live."

"The first appearance of the throat distemper was at Kingston, an inland town of New England, about the year 1735; and as this town has no foreign trade, it may be concluded that the disease was not imported. It spread from thence and moved gradually westward, so that it did not reach Hudson's river till two years afterward. It continued some time on the east side of Hudson's river before it passed to the west, and appeared first in those places to which the people of New England resorted for trade, and in the places, through which they travelled. It continued to move westerly till, I believe, it has at last spread over all the British colonies on the continent."

"Though what I have mentioned seems evidently to show that the disease was propagated by infection, yet it did not spread in the same manner contagious distempers usually do; for children and young people only were subject to it, with a few exceptions of some few above twenty or thirty, and a very few old people who died of it,neither did it spread equally in all places that were equally exposed to the infection. The poorer sort of people were more liable to have this disease than those who lived well with all the conveniences of life. It has been more fatal in the country than in the great towns. People of a scorbutic habit were most subject to it, and they who fed on pork or lived in wet and low grounds. In some places only a few persons or families were seized, while in others all escaped. In some families it passed like a plague through all their children, in others only one or two were seized with it. Some were seized with it at such a distance from the infected, that it could not be conceived in what manner they could receive the disease by infection. Some families had the disease mildly, while others, in the same place and at the same time, had a most violent sort."

*In a letter of Dr. J. C. Warren reference is made to "a treatise of Dr. Douglass, of Boston, on this subject" of the date of 1736. I have not been able to meet with the work.

"Ever since it came into the part of the country where I live (now about 14 years) it frequently breaks out in different families and places, without any previous observable cause; but it does not spread as it did at first: sometimes a few only have it in a considerable neighborhood. It seems as if some seed or leaven, or secret cause, remained wherever it goes; for I hear of the like observation in other parts of the country.

"In different years, and different persons the symptoms are various. In some seasons it has been accompanied with miliary eruptions all over the skin; and at such times, the symptoms about the throat have been mild, and the disease generally without danger, if not ill treated."

Such is the earliest record I have seen, by an actual intelligent observer, of the disease on this continent; and it tallies accurately in all respects with more recent observation. As now, so then, it varied in the violence of its attack, in the absence or presence of eruption, and the degree of mortality; and then, as now the different forms prevailed simultaneously and separately, and there were periods of epidemic prevalence with occasional cases occuring sporadically. The same uncertainty marks its commencement and mode of propagation.

The next earliest notice of the disease on this continent by a medical author that has met my eye, is an exceedingly interesting manuscript tract on the subject, by Dr. John Kearsley of this city, now in the possession of Dr. George W. Norris. He says: "In the spring, summer and autumn of 1746," nearly ten years from the time of its first appearance at Kingston, "and indeed some part of the winter of the same year, this disease since called, by the learned Huxham, Fothergill and others, the angina maligna, or the putrid and ulcerous sore throat, prevailed in this and the neighboring provinces, and spread itself with mortal rage in opposition to the united endeavors of the faculty. Like to some new diseases, till their nature and constitution are known, it swept away all before it. It baffled every attempt to stop its progress, and seemed by its dire effects to be more like the drawn sword of vengeance to stop the growth of the colonies, than the real progress of a disease. In the New England Governments, as their annals no doubt will show, the stroke was felt with great severity. Villages were almost depopulated, and parents were left to bewail the loss of their tender offspring, till Heaven, at last, whose almighty power we all must own, graciously checked its baneful influence. This disease as it then appeared, and since within these few years, had most of those symptoms to characterize it which those learned gentlemen, we have mentioned, have handed down to us. With us it generally affected children, or those under puberty, whose lax solids and spongy habits were more fitted to receive the floating miasmata of a putrid atmosphere, than those whose textures are more solid. It also affected those who lived in low, wet, marshy places, more than those who lived in higher situations. It happened with us, as it did with them in England, after much cold wet weather, succeeded by heat." At this period we find the burials in the grounds of Christ Church, rising to a number even greater than those in the year 1741, in which a pestilence, the nature of which is not recorded, prevailed in the city, and by the law that more than one form of epidemic disease is never prevalent at one time, we are justified in attributing this great increase of mortality to scarlet fever. I have derived these statements respecting the prevalence of the disease in this country, chiefly from Webster's elaborate work on Epidemic Diseases, which exhibits the dates at which it prevailed, and the general mortality of each year, without giving any minute detail.

That this disease should affect both sexes equally might be reasonably inferred from the want of any circumstance, which should cause one to be more liable to its influence, than another. From partial observations, varying conclusions on this point have been reached by different authors. I need not now inform you that no accurate results can be obtained by the most carefully arranged tables, unless the observations be conducted on an extensive field. Unhappily for humanity, as well as for the scientific character of our country, the efforts made by the medical profession to arouse attention to the importance of an uniform system of Registration, have not yet accomplished an object so earnestly to be desired. We are, therefore, compelled to resort for extended statistical statements to foreign states. Thus while the private observation of Dr. Tweedie in London leads him to assert, that it is more common in girls; and from the same source, Rilliet and Barthez reach the conclusion, that it is more common in boys, and Dr. J

F. Meigs thinks it "probable that while under puberty, it attacks the two sexes with about equal frequency, and after that age it is most common in females," the reports of the Register-General of England and Wales, quoted by Dr. Gregory, show that in 1838, it destroyed, in London, 747 males, and 777 females; in 1839, 1214 males, and 1258 females. Throughout England and Wales (exclusive of the metropolis) in 1840, 8927 males, and 8935 females. Though in each of these statements there is an excess of females over males, it is so small a fraction of the whole number, that it cannot fairly be ascribed to any especial liabity of one sex to the complaint. If the one sex were more subject than the other to the disease, the difference would be more marked. Extended observation would in all probability change the result.

But while I believe sex exerts no influence over this disease, the relations which *age* bears to it are very important. It is decidedly a disease of childhood, and that, not because all persons are exposed to it in the earlier years of life, and those who are susceptible take it then and lose their liability, but because there is something in the condition of the system during infancy and adolescence, which renders those periods of life especially subject to the disease. Thus you will find that Dr. Kearsley and Mr. Colden both refer to the fact, that the force of the epidemics they witnessed fell upon children, and attempt to account for this preference. It must not be overlooked, that at the date of the visitation they witnessed, the adult inhabitants of the colonies had passed through infancy and youth without exposure, so that their susceptibility could not have been exhausted by a previous attack. That adults are liable to it in rare instances, with great, even fatal severity, cannot be denied. The general expression is, however, correct, that Scarlet Fever is a disease of infancy and childhood.

(To be Continued.)

